# Anti-SARS-CoV-2 antibody dynamics after primary vaccination with two-dose inactivated whole-virus vaccine, heterologous mRNA-1273 vaccine booster, and Omicron breakthrough infection in Indonesian health care workers

**DOI:** 10.1186/s12879-024-09644-y

**Published:** 2024-08-01

**Authors:** Suwarti Suwarti, Gilbert Lazarus, Sabighoh Zanjabila, Robert Sinto, Fransiska Fransiska, Theresia Deborah, Dwi Oktavia, Junaidah Junaidah, Santayana Santayana, Henry Surendra, Jeng Yuliana, Herlina Pardosi, Nunung Nuraeni, Saraswati Soebianto, Novi Dwi Susilowati, Decy Subekti, Ariel Pradipta, J. Kevin Baird, Le Van Tan, Susanna Dunachie, Anuraj H. Shankar, Erni J. Nelwan, Raph L. Hamers, SEACOVARIANTS Consortium, Nguyen To Anh, Nguyen Thi Thu Hong, Truong Hoang Chau Truc, Nguyen Thi Han Ny, Do Duong Kim Han, Le Kim Thanh, Lam Anh Nguyet, Cao Thu Thuy, Le Nguyen Truc Nhu, Tran Tan Thanh, Lam Minh Yen, Vu Thi Ty Hang, Pham Tieu Kieu, Vo Tan Hoang, Nguyen Thi Thao, Mary Chambers, Vu Duy Thanh, Tran Chieu Hoang, C. Louise Thwaites, Guy Thwaites, H. Rogier van Doorn, Trinh Son Tung, Juthathip Mongkolsapaya, Gavin Screaton, Aiete Dijokaite-Guraliuc, Raksha Das, Chang Liu, Piyada Supasa, Muneeswaran Selvaraj, Susanna J. Dunachie, Paul Klenerman, E. Yvonne Jones, David I. Stuart, Barbara Kronsteiner-Dobramysl, Martha Zewdie, Priyanka Abraham, Jennifer Hill, Yanie Tayipto, Isana Paramita, Wang Lin-Fa, Tan Chee Wah, Yap Wee Chee, Lim Beng Lee, Suwarti, Eva Simarmata, Ragil Dien, Wanwisa Dejnirattisai, Warangkana Chantima, Narisara Chantratita, Prapassorn Poolchanuan, Vichapon Tiacharoen, Adul Dulsuk, Sophon Iamsirithaworn, Nick Day, Phaik Yeong Cheah, Tassawan Poomchaichote, Kanpong Boonthaworn, Nghiem My Ngoc, Alba Grifoni, Alessandro Sette

**Affiliations:** 1 Oxford University Clinical Research Unit Indonesia, Faculty of Medicine Universitas Indonesia, Jakarta, Indonesia; 2 Infectious Disease and Immunology Research Cluster, Indonesian Medical Education and Research Institute, Jakarta, Indonesia; 3 Faculty of Medicine, Universitas Indonesia, Jakarta, Indonesia; 4 Division of Tropical and Infectious Diseases, Department of Internal Medicine, Cipto Mangunkusumo National Hospital, Jakarta, Indonesia; 5 St Carolus Hospital, Jakarta, Indonesia; 6 Jakarta Health Office, Ministry of Health Republic of Indonesia, Jakarta, Indonesia; 7 Monash University, Tangerang, Indonesia; 8 Nuffield Department of Medicine, Centre for Tropical Medicine and Global Health, University of Oxford, Oxford, UK; 9 Genomik Solidaritas Indonesia Lab, Jakarta, Indonesia; 10 Department of Biochemistry and Molecular Biology, Faculty of Medicine, Universitas Indonesia, Jakarta, Indonesia; 11 Oxford University Clinical Research Unit, Ho Chi Minh City, Vietnam; 12 Mahidol-Oxford Tropical Medicine Research Unit, Mahidol University, Bangkok, Thailand

**Keywords:** SARS-CoV-2, COVID-19, Humoral immunity, CoronaVac, mRNA-1273

## Abstract

**Background:**

Data on the dynamics and persistence of humoral immunity against SARS-CoV-2 after primary vaccination with two-dose inactivated vaccine (CoronaVac) are limited. This study evaluated the sequential effects of prior infection, heterologous boosting with mRNA-1273 (Moderna), and the occurrence of Omicron vaccine-breakthrough infection (VBI) thereafter.

**Methods:**

We evaluated anti-spike IgG (Abbott) and neutralising (cPASS/GenScript) antibody (nAb) titers up to one year after mRNA-1273 boost in two-dose-CoronaVac-primed Indonesian healthcare workers (August 2021-August 2022). We used linear mixed modeling to estimate the rate of change in antibody levels, and logistic regression to examine associations between antibody levels and VBI.

**Results:**

Of 138 participants, 52 (37.7%) had a prior infection and 78 (56.5%) received an mRNA-1273 booster. After two-dose CoronaVac, antibody titers had significantly declined within 180 days, irrespective of prior infection. After mRNA-1273 booster, anti-spike IgG (1.47% decline/day) and Omicron B.1.1.529/BA.2 nAbs declined between day 28–90, and IgG titers plateaued between day 90–360. During the BA.1/BA.2 wave (February–March 2022), 34.6% (27/78) of individuals experienced a VBI (median 181 days after mRNA-1273), although none developed severe illness. VBI was associated with low pre-VBI anti-spike IgG and B.1.1.529/BA.2 nAbs, which were restored post-VBI.

**Conclusions:**

mRNA-1273 booster after two-dose CoronaVac did not prevent BA.1/BA.2 VBI. Periodic vaccine boosters may be warranted against emerging SARS-CoV-2 variants.

**Supplementary Information:**

The online version contains supplementary material available at 10.1186/s12879-024-09644-y.

## Key points

An ancestral-strain mRNA-1273 (Moderna) vaccine boost after two-dose inactivated virus vaccine (CoronaVac) did not prevent Omicron BA.1/BA.2 breakthrough infections in Indonesian healthcare workers, and were associated with declined anti-spike and neutralizing antibodies.

**Supplementary Information:**

The online version contains supplementary material available at 10.1186/s12879-024-09644-y.

## Introduction

Coronavirus disease 2019 (COVID-19), caused by the severe acute respiratory syndrome coronavirus-2 (SARS-CoV-2), has significantly impacted global economies, societies, and public health. By February 1st, 2024, Indonesia, the world’s fourth most populous country with 275 million people, reported 6.8 million cases and 161.000 deaths. Indonesian frontline healthcare workers (HCWs) were disproportionately impacted during the first 18 months of the pandemic, with an estimated mortality rate of approximately 1.7 deaths per 1000, which is five times higher than that of the general population [1].

Vaccination is a crucial global strategy to control the COVID-19 pandemic, with several vaccines proving to be safe and effective in preventing COVID-19-related hospitalization and death. Data primarily from studies on viral vector and mRNA vaccines indicate that humoral responses wane within 6 months after the second dose [2, 3] and there is a time-progressive increase in vaccine-breakthrough infection (VBI), particularly with the Omicron variant (B.1.1.529) and its subvariants [4]. However, a third vaccine dose significantly restores antibody levels [5].

CoronaVac (CV) (SinoVac Life Sciences Co. Ltd., Beijing, China), an inactivated whole-virus vaccine, has been the predominant vaccine used in Indonesia’s mass vaccination campaign. Despite reports of high effectiveness [6], data suggest that CV has lower immunogenicity compared to viral vector and mRNA vaccines [7], with a marked decline in neutralizing antibody titers within a few months [8–10].

To date, most of the global population has developed heterogenous immune histories from various exposures to infection with different viral variants, and diverse vaccination types and regimens, referred to as “hybrid immunity” [11, 12]. Both infection and vaccination can induce strong, but short-lived, immune protection against reinfection [13]. While many countries have implemented third or even successive vaccine doses, there is limited data on the durability and dynamics of neutralizing antibodies for primary regimens based on inactivated vaccines, including the effects of prior infection, heterologous vaccine boosters, and VBI.

In a longitudinal adult cohort in Jakarta, Indonesia, we described the dynamics and persistence of spike-specific IgG and neutralizing antibodies against SARS-CoV-2 variants in adults who had received two-dose CoronaVac primary vaccination, and examined the sequential effects of prior infection, a third heterologous vaccine dose (booster) of mRNA-1273 (Moderna), and the occurrence of Omicron VBI thereafter. For comparison, the cohort also included a sub-group of participants who had only received two-dose CoronaVac primary vaccination.

## Methods

### Design and population

The Indonesia Vaccine Immunity & Infection Evaluation (INVITE) study is a longitudinal observational cohort, as described elsewhere [12]. Briefly, we consecutively enrolled HCWs (aged ≥ 18 years) on the day they received their 100-μg mRNA-1273 third dose (between August 6th and October 4th, 2021), after having completed the two-dose CoronaVac primary regimen at least 6 months prior (denoted as CV-CV-mRNA vaccinees). We also enrolled individuals from the general population (aged ≥ 12 years) on the day they received the first dose of their two-dose CoronaVac primary regimen (between November 16th, 2021 and June 10th, 2022) (denoted as CV-CV vaccinees). The present analysis included all participants for whom at least two serial stored serum samples were available, including the pre-vaccine (day 0) and at least one post-vaccine dose (timepoints day 28, 90, 180 for all; and day 360 and at time of VBI for CV-CV-mRNA vaccinees) (Figure S1, Table S1).

The outcomes of interest were:

1. Titers and the rate of change over time of anti-Spike Receptor Binding Domain IgG, and neutralizing antibody inhibition (nAbs), comparing CV-CV-mRNA and CV-CV vaccinees with or without history of prior infection (PI versus noPI); PI was defined as, for CV-CV vaccinees, presence of anti-SARS-CoV-2 nucleocapsid protein, or a history of rapid antigen test or PCR-confirmed SARS-CoV-2 before first vaccine dose, and, for CV-CV-mRNA vaccinees, a history of rapid antigen test or PCR-confirmed SARS-CoV-2 infection before third dose vaccine.
2. Occurrence of VBI after third (mRNA-1273) vaccine dose, defined as a PCR-confirmed SARS-CoV-2 infection at least 14 days post-vaccination. For this analysis, we compared participants with VBI (*n* = 27) with randomly selected participants without a history of VBI (noVBI, *n* = 50), matched for sex, age group and calendar month.

This study is reported as per Strengthening the Reporting of Observational Studies in Epidemiology guidelines.

### Procedures and assays

Study data were collected and managed using a REDCap electronic data capture tool. To detect SARS-CoV-2 infection, we adopted the following test strategies: 1) all participants were asked to undergo either RT-PCR or a rapid antigen test in case of exposure to an infected individual or COVID-19-like symptoms); 2) all participants who reported any COVID-19-like symptoms at a study visit were RT-PCR tested; 3) During the successive SARS-CoV-2 surges, HCW participants were routinely tested every month using either RT-PCR (January to April 2022) or rapid antigen testing (May to December 2022).

RT-PCR-positive specimens were submitted for SARS-CoV-2 whole genome sequencing at Genomik Solidaritas Laboratory (GSI) in Jakarta. Anti-nucleocapsid protein antibody seropositivity was measured using the SARS-CoV-2 NP IgG ELISA Kit (My Biosource, MBS398004) following the kit manual instructions; sera were incubated in a recombinant nucleocapsid protein pre-coated well followed by HRP-conjugated solution at room temperature for an hour. Absorbance at 450 nm was measured with a ratio above 1.1 considered positive. Titers of anti-S IgG were determined using the chemiluminescent microparticle immunoassay (CMIA) SARS-CoV-2 IgG II Quant assay (Abbott Laboratories, Abbott Park, IL, US) on the Architect i2000sr platform, in accordance with the manufacturers’ instructions, and expressed in WHO International Standard binding antibody units (BAU)/mL using the manufacturer’s conversion factor (1 BAU/mL = 0.142 × arbitrary units[AU]/mL) and seropositivity defined as ≥ 7.1 BAU/mL. nAbs were measured using SARS-CoV-2 Surrogate Virus Neutralization Test (cPASS, GenScript, USA) against SARS-CoV-2 Spike protein RBDHRP wild-type (L00847-A), Delta (Z03614), Omicron B.1.1.529 (Z03730) and Omicron BA.2 (Z03741) according to the manufacturers’ instruction; sera were diluted 1:20 with diluent assay and pre-incubated with SARS CoV-2 HRP-RBD in a 1:1 volume ratio at 37 °C for 30 min. The mixture was then added to a capture plate pre-coated with hACE2 protein. Absorbance at 450 nm was measured, which is inversely proportional to the titer of the anti-SARS-CoV-2 neutralizing antibodies. nAb detection was expressed as the signal inhibition (SI, %), with a cut-off of ≥ 30%.

### Statistical analysis

Descriptive statistics included proportions for categorical variables and medians and interquartile ranges (IQRs) for continuous variables. Correlations of log_10_-transformed titers were expressed using the Spearman’s rank correlation coefficient tests (r_s_). We used Kruskal–Wallis H with Dunn’s post-hoc tests to perform comparisons of anti-S IgG and nAb titers before and after the third mRNA vaccine dose, and pairwise comparisons between groups. *P*-values were adjusted for multiple comparisons by Benjamini-Hochberg’s method.

To examine antibody waning, we fit a linear mixed model comprising a fixed effect linear decline from 28 days (peak) after vaccination onward and 28-day peak anti-S IgG titers as a random effect, modelled as a constant. Separate models were run for second and third vaccine doses, and for models with and without VBI, where we added a knot at day 90 and 180 to reflect the inflection point of the anti-S IgG titer trends.

To examine the association between most recent anti-S IgG and nAb levels prior VBI and the occurrence of VBI, we fit a complete-case analysis logistic regression model, adjusted for age and PI. VBI participants were matched with noVBI participants based on time since third vaccine dose and calendar month.

All analyses were done using Stata/IC 15.1 (StataCorp, College Station, TX, USA) and visualized using R version 4.2.2 (R Foundation for Statistical Computing, Vienna, Austria) and GraphPad Prism10. A two-sided *P* < 0.05 was considered significant.

## Results

### Participant characteristics

Of 1117 cohort participants who received two-dose CV primary vaccination, we included a randomly selected subset of 138 (12.4%) individuals for whom at least a pre- and a post-vaccination sample was available, comprising 60 CV-CV and 78 CV-CV-mRNA vaccinees (Figure S1, Table 1). 52 (37.7%) had PI and 78 (56.5%) received a third (mRNA-1273) vaccine dose. Median age was 34 years (IQR26-49) and 78 (56.5%) were women. Table S2 summarizes the key characteristics of the included participants compared to the whole cohort, and Table S1 the number of included participants per timepoint. Figure S2 shows the cohort timeline from December 2020 through January 2023, in the context of the successive epidemic waves in Indonesia.

**Table 1:** Participant characteristics at enrolment

Characteristic	Total (*N* = 138)	CV-CV vaccinees^1^ (*N* = 60)		CV-CV-mRNA vaccinees^2^ (*N* = 78)	
		**No prior infection (*N* = 25)**	**Prior infection** **(*N* = 35)**	**No prior infection** **(*N* = 61)**	**Prior infection** **(*N* = 17)**
Sex					
Female	78 (56.5)	10 (40.0)	12 (34.3)	45 (73.8)	11 (64.7)
Male	60 (43.5)	15 (60.0)	23 (65.7)	16 (26.2)	6 (35.3)
Age (median, IQR) – yrs	34.0 (26.0–49.0)	37.0 (26.0–48.0)	40.0 (27.0–55.0)	30.0 (26.0–48.0)	31.0 (27.0–37.0)
< 30	56 (41.3)	9 (36.0)	12 (34.3)	29 (47.5)	6 (35.3)
30–49	52 (37.0)	10 (40.0)	9 (25.7)	24 (39.3)	8 (47.1)
≥ 50	30 (21.7)	6 (24.0)	14 (40.0)	8 (13.1)	3 (17.7)
Any comorbidity	63 (45.7)	14 (56.0)	18 (51.4)	22 (36.1)	9 (52.9)
Obesity	44 (31.9)	6 (24.0)	11 (31.4)	20 (67.2)	7 (41.2)
Hypertension	15 (10.9)	4 (16.0)	4 (11.4)	3 (4.9)	4 (23.5)
Diabetes mellitus	8 (5.8)	4 (16.0)	2 (5.7)	1 (1.6)	1 (5.9)
Chronic lung disease	4 (2.9)	2 (8.0)	2 (5.1)	0 (0.0)	0 (0.0)

*Abbreviations*: *IQR* interquartile range

Prior infection (PI) was defined as, for CV-CV vaccinees, presence of anti-SARS-CoV-2 nucleocapsid protein, or a history of rapid antigen test or PCR-confirmed SARS-CoV-2 before first vaccine dose, and, for CV-CV-mRNA vaccinees, a history of rapid antigen test or PCR-confirmed SARS-CoV-2 infection before third dose vaccine. The interval between second and third vaccine dose (CV-CV-mRNA) was median 185 days (IQR168–195)

^1^General population participants who received CoronaVac primary vaccination only (CV-CV; 2dose) and were followed up for up to 180 days post-vaccination

^2^Healthcare workers who received mRNA-1273 after CoronaVac primary vaccination (CV-CV-mRNA; 3dose) and were followed for up to 360 days post-vaccination

### Dynamics of anti-spike IgG titers and neutralizing antibodies after two or three vaccine doses

Whereas pre-vaccine (Day 0) anti-S IgG titers showed strong correlations with all tested nAbs (i.e. Wild-type, Delta, Omicron B.1.1.529 and BA.2) among both CV-CV and CV-CV-mRNA vaccinees (each r_s_ > 0.7), anti-S IgG titers on Day 28 after the second vaccine dose showed strong correlations with wild-type, Delta and BA.2 nAbs (each r_s_ > 0.8) and moderate correlations with B.1.1.529 nAbs (r_s_ = 0.541) in CV-CV vaccinees; and moderate correlation with B.1.1.529 nAbs (r_s_ = 0.440) and weak correlation with BA.2 nAbs (r_s_ = 0.303) in CV-CV-mRNA vaccinees (wild-type and Delta not estimable) (Figure S3).

**Fig. 1:**
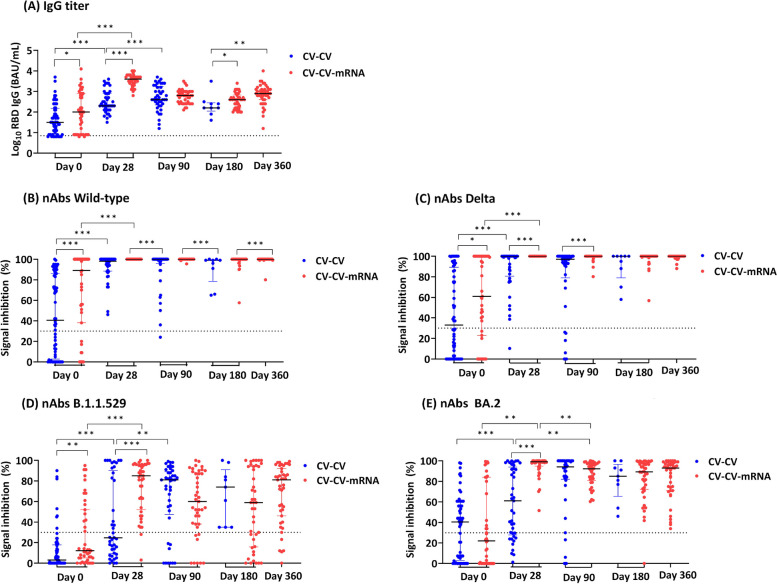
Antibody responses against SARS-CoV-2 variants in CV-CV and CV-CV-mRNA vaccinees The figure shows data separately for the CV-CV vaccinees (blues dots) and the CV-CV-mRNA vaccinees (red dots). Serum anti-spike IgG titers (A) and neutralizing antibodies (nAbs) against SARS CoV-2 wild-type (B), Delta (C), Omicron B.1.1.529 (D) and BA.2 (E) were measured: i) Pre-vaccination (Day 0), i.e. before the first dose in CV-CV vaccinees (n=60) and before third dose in CV-CV-mRNA vaccinees (n=42) ii) Post-vaccination day 28 (CV-CV n=41 and CV-CV-mRNA n=40), day 90 (CV-CV n=41 and CV-CV-mRNA n=40), day 180 (CV-CV n=9 and CV-CV-mRNA n=40), and day 360 (CV-CV-mRNA n=43). Dashed line represents the seropositivity of IgG titer [≥7.1 BAU/mL = 0.85 log10 BAU/mL] and cut-off value for presence of neutralizing antibodies [signal inhibition >30%]. P-values were derived from Kruskal-Wallis H followed by Dunn’s post-hoc tests, adjusted by Benjamini-Hochberg method for multiple comparisons. *, p<0.05; **, p<0.01; ***, p<0.001 Abbreviations: BAU, binding antibody units; CV, CoronaVac; mRNA, mRNA-1273 (Moderna)

After the second (CV) vaccine dose, the day-28 anti-S IgG peak titer was 1.6-fold lower than after the third (mRNA-1273) vaccine dose (median 2.3 [IQR2.1–2.9] vs 3.6 [3.4–3.7] log_10_ BAU/mL vs; *p* < 0.001). On day 28 after the second (CV) vaccine dose, wild-type nAbs were detected in all participants, Delta nAbs were detected in all but one individual, whereas B.1.1.529 nAbs were not detected in 23 individuals (43.9% [18/41] seropositivity) and BA.2 nAbs in 12 individuals (70.7% [29/41] seropositivity). Between day 28 and 90 after the second (CV) vaccine dose, B.1.1.529 (78.0% [32/41] seropositivity) and BA.2 (87.8% [36/41] seropositivity) nAbs remained reduced, and subsequently increased between day 90 and 180 (100% [9/9] seropositivity each) (Fig. 1, Table S3). This latter finding possibly reflects unrecorded virus exposure during the BA.1/BA.2 wave in February–March 2022 (Figure S2).

After the third (mRNA-1273) vaccine dose, we observed a significant day-28 peak in anti-S IgG and nAbs against all variants, compared to before the third dose (Fig. 1, Table S3). On day 28 wild-type and Delta nAbs and BA.2 nAbs (100% [40/40] seropositivity and median SI 99.0% [IQR95.0–100.0]) were detected in all participants (100% [40/40] seropositivity), whereas B.1.1.529 nAbs were not detected in two individuals (95.0% [38/40] seropositivity). Between day 28 and 90 after the third (mRNA-1273) dose, anti-S IgG titers had decreased significantly (from median 3.6 [IQR3.4–3.7] to 2.8 [2.4–3.0] log_10_ BAU/mL; *p* < 0.001), and then reached a plateau between day 90 and 360 days (*p* = 0.161). Whereas Wild-type and Delta nAbs persisted up to day 360 (100% [40/40] seropositivity), Omicron nAbs had declined significantly between day 28 and 90 for B.1.1.529 and BA.2 (Fig. 1, Table S3).

### Effect of prior SARS-CoV-2 infection on antibody responses

Compared with individuals without PI at the time of the first CV dose, individuals with PI had 2.1-fold higher anti-S IgG titers (median 1.9 [IQR1.5–2.6] vs 0.9 [0.8–1.25] log_10_ BAU/mL; *p* < 0.001) and higher nAbs against all variants, i.e. Wild-type (80.0% [28/35] vs 28.0% [7/25] seropositivity; *p* < 0.001); Delta (74.3% [26/35] vs 16.0% [4/25] seropositivity; *p* < 0.001); B.1.1.529 (20.0% [7/35] vs 0.0% [0/25] seropositivity; *p* < 0.001); and BA.2 (77.1% [27/35] vs 28.0% [7/25] seropositivity; *p* < 0.001) (Figure S4, Table S4).

Similarly, compared with individuals without PI at the time of the third (mRNA-1273) vaccine dose, individuals with PI had 2.1-fold higher anti-S IgG titers (median 2.7 [IQR2.0–2.3] vs 1.3 [0.9–2.2] log_10_ BAU/mL; *p* = 0.002) and nAbs against wild-type (100% [16/16] seropositivity vs 61.5% [16/26] seropositivity; *p* = 0.002); Delta (93.8% [15/16] vs 57.7% [15/26] seropositivity; *p* = 0.002); and BA.2 (68.8% [11/16] vs 23.1% [6/26]; *p* = 0.006); but there was no statistical difference for B.1.1.529 nAbs (50.0% [8/16] vs 23.1% [7/26] seropositivity; *p* = 0.329) (Figure S4, Table S4).

By contrast, on day 360 of follow-up after the third (mRNA-1273) vaccine dose, there were no longer statistically significant differences between participants with or without PI, both in regard to anti-S IgG titers (*p* = 0.056) and nAbs against wild-type (*p* = 0.077), Delta (*p* = 0.333), B.1.1.529 (*p* = 0.488), and BA.2 (*p* = 0.377) (Figure S4, Table S4).

### Omicron breakthrough infections after the third mRNA vaccine dose

**Fig. 2:**
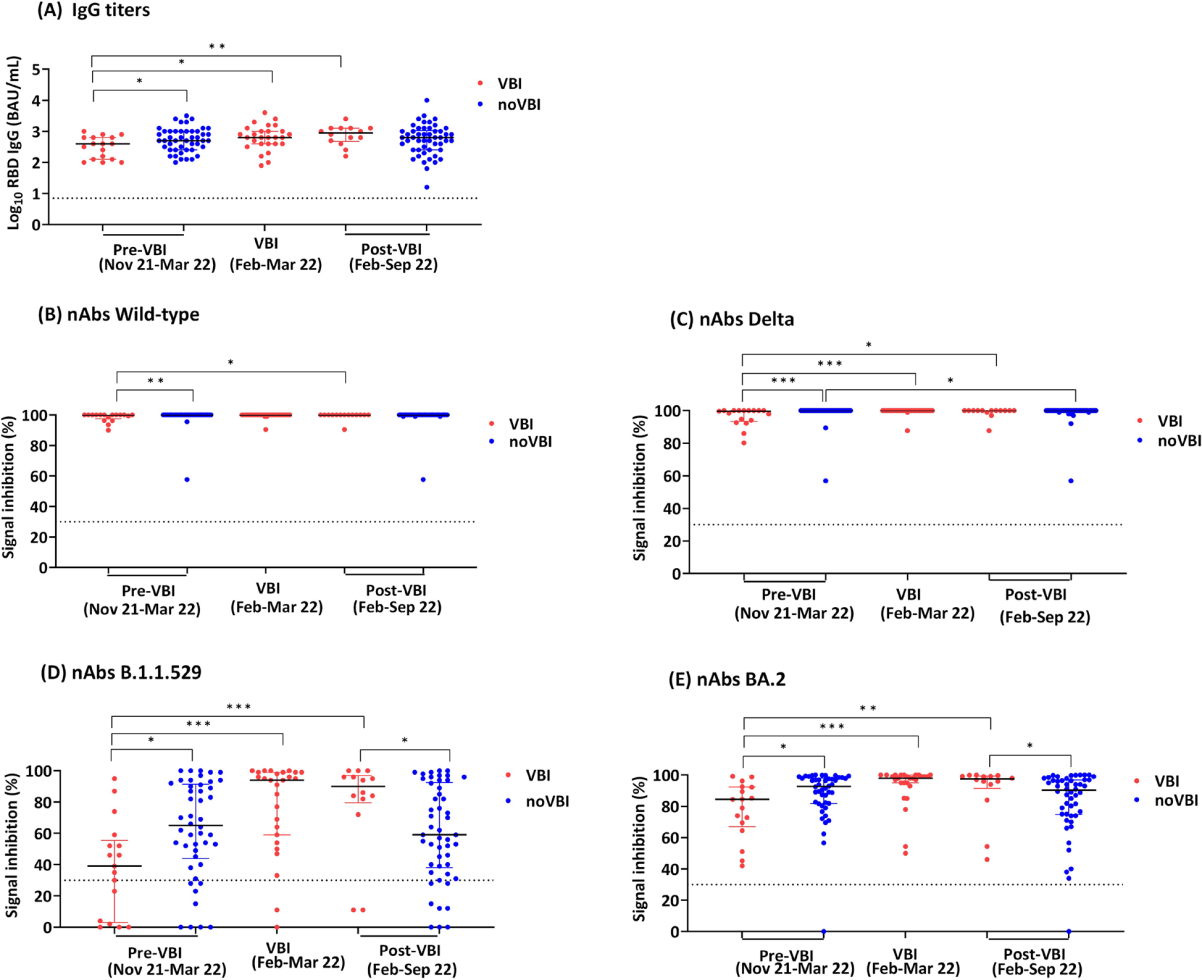
Antibody responses against SARS-CoV-2 in CV-CV-mRNA vaccinees before, during and after Omicron breakthrough infection, compared to those who never experienced breakthrough infection Figure shows anti-S IgG titers (A) and neutralizing antibodies (nAbs) against wild-type (B), Delta (C), Omicron B.1.1.529 (D) and Omicron BA.2 (E) in participants who experienced an Omicron  BA.1/BA.2 vaccine breakthrough infection (VBI; red dots, n=27) and those who did not (noVBI;blue dots, n=56). VBI (red dots) were those who had a PCR-confirmed infection at least 14 days after the third dose (CV-CV-mRNA vaccinees). The median time of VBI occurrence was 181 days (IQR170-202) since the third mRNA vaccine dose. NoVBI participants (blue dots) were those who had never experienced a VBI during or prior the Omicron wave (February-March 2022). Pre-VBI antibody levels represent the most recent measurement prior to VBI occurrence (median 55 days [IQR33-88] before VBI). Post-VBI antibody level represents the most recent measurement after VBI occurrence (median 101 days [IQR39-175] after VBI). P-values were derived from Kruskal-Wallis H followed by Dunn’s post-hoc tests, adjusted by Benjamini-Hochberg method for multiple comparisons. *, p<0.05; **, p<0.01; ***, p<0.001 Abbreviations: BAU, binding antibody units; CV, CoronaVac; mRNA, mRNA-1273 (Moderna)

After the third (mRNA-1273) vaccine dose, 27 (34.6%) of 78 individuals experienced a VBI, mostly during the BA.1/BA.2 wave in February–March 2022 (Table S5), at a median of 181 (170–202) days after the mRNA-1273 boost (Fig. 2). None of the VBI cases were severe, four cases were asymptomatic, 22 cases were mild, and one case was moderate. Whole genome sequencing demonstrated BA.1 (*n* = 10), BA.2 (*n* = 12) and BE (*n* = 1), and from 4 samples the virus could not be sequenced.

After adjusting for age and prior COVID-19 history, VBI occurrence was statistically significantly associated with lower pre-VBI antibody titers (median 55 days [IQR33-88] before VBI), specifically anti-S IgG (aOR5.42 for each log_10_ titer decrease [95%CI1.08–27.21], *p* = 0.040), B.1.1.529 nAbs (1.93 increased odds for each 25% SI decrease [1.18–3.14], *p* = 0.009) and BA.2 nAbs (2.13 increased odds for each 25% SI decrease [0.95–4.79], *p* = 0.068), but not associated with Wild-type or Delta nAbs (Table 2).

**Table 2:** Association of most recent antibody levels with subsequent Omicron breakthrough infection in CV-CV-mRNA vaccines

Variables	Unadjusted			Adjusted		
	**OR**	**95%CI**	***P*-value**	**OR**	**95%CI**	***P*-value**
Anti-S IgG (per log_10_ titer decrease)	**5.31**	**1.07–26.36**	**0.041**	**5.42**	**1.08–27.21**	**0.040**
Neutralizing antibodies (per 25% SI decrease)						
Wild-type	1.46	0.15–14.66	0.749	1.47	0.14–15.31	0.746
Delta	4.25	0.44–41.52	0.213	4.25	0.44–40.88	0.210
Omicron B.1.1.529	**1.88**	**1.17–3.03**	**0.009**	**1.93**	**1.18–3.14**	**0.009**
Omicron BA.2	2.08	0.93–4.67	0.076	2.13	0.95–4.79	0.068

*Abbreviations*: *CI* confidence interval, *OR* odds ratio, *SI* signal inhibition, *VBI* vaccine breakthrough infection

The table shows results of the complete-case (*N* = 63) logistic regression model estimating the association between antibody levels and subsequent Omicron BA.1/BA.2 breakthrough infection, unadjusted (left) and adjusted for age and prior history of COVID-19 infection (right), with antibody levels analyzed iteratively to prevent collinearity between variables. We included the most recent antibody titers measured prior to the occurrence of Omicron breakthrough infection (median 55 days [IQR33-88] before VBI). Median time since latest (third) vaccine dose was 181 days (IQR170-202)

Subsequently, participants who had experienced a VBI developed significantly higher post-VBI nAbs levels against Omicron variants (median 101 days [IQR39-175] after VBI), compared to those who had never had a VBI, i.e. B.1.1.529 (median SI 90.0% [IQR79.5–97.0] vs 59.0% [38.0–92.5]; *p* = 0.013) and BA.2 (97.5% [91.5–99.3] vs 90.2% [74.7–96.9]; *p* = 0.021) (Fig. 2, Table S5).

### Changes of anti-spike IgG titers after second and third vaccine dose

In a linear mixed model that adjusted for age and monthly new COVID-19 cases in Jakarta, PI was associated with higher anti-S IgG titers on day-28 (peak) after primary CV vaccination compared to not having a history of PI (least square mean [LSM] 2.49 [95%CI2.32, 2.68] vs 2.11 [1.85, 2.38] log_10_ BAU/mL). After the second (CV) vaccine dose, anti-S IgG titers increased between day 28 and 90 (0.74% increase per day [0.39% to 1.09%], *p* < 0.001) (due to possible unrecorded Omicron exposure), and declined thereafter between day 90 and 180 (0.44% decline per day [0.11% to 0.76%], *p* = 0.008). PI had no significant effect on anti-S IgG decline between day 90 and 180 after two-dose CV primary vaccination (0.64% decline per day [0.24% to 1.04%; *p* = 0.002 for PI participants vs 0.25% decline per day [-0.70% to 0.20%, *p* = 0.272) for noPI participants (Table S6).

**Fig. 3:**
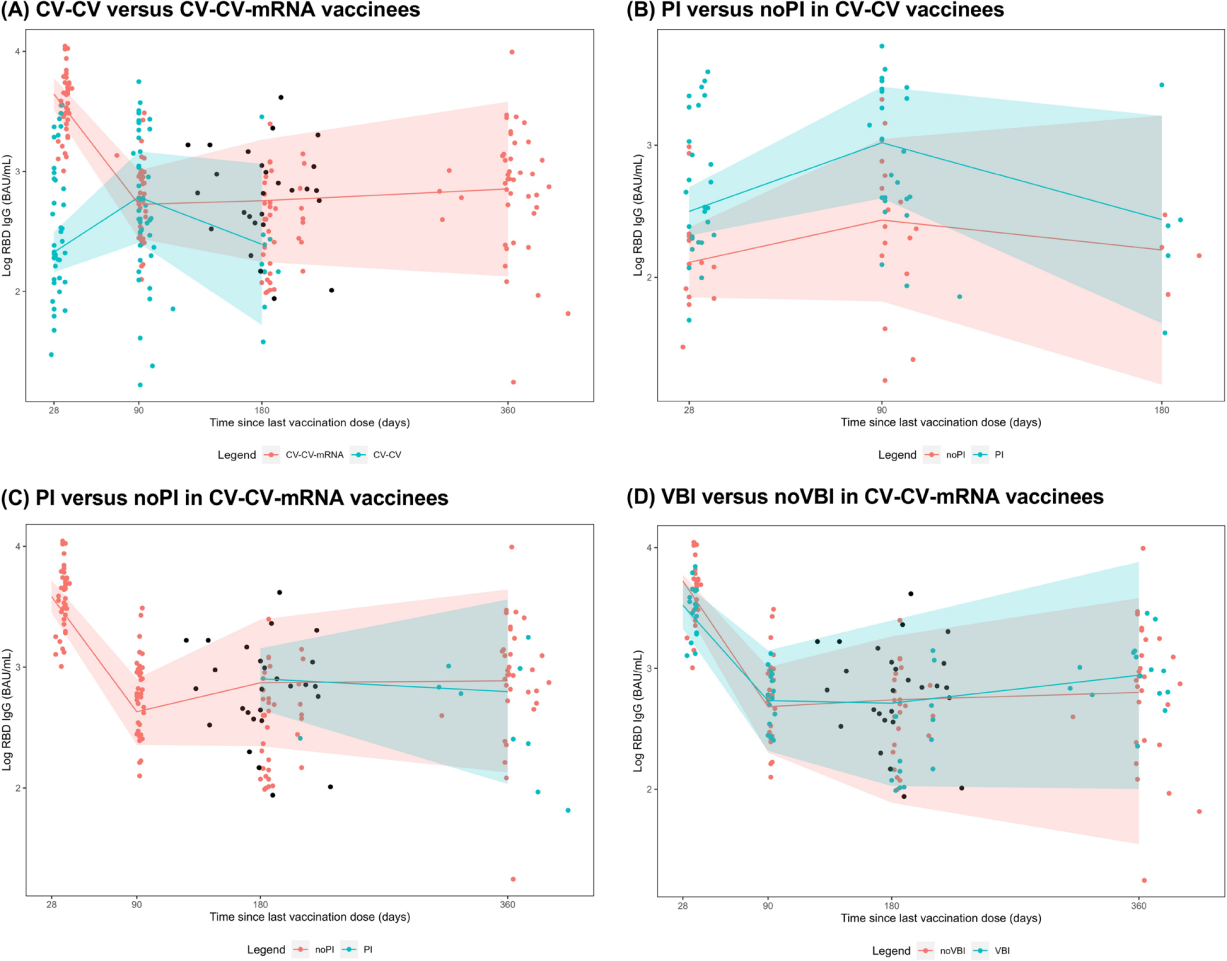
Dynamic changes in anti-spike IgG titers after second (CV) and third (mRNA-1273) vaccine dose Figure shows dynamic changes in anti-spike IgG titers between: (A) CV-CV and CV-CV-mRNA vaccinees after the latest vaccine dose on day 28 (n=41 and n=40), day 90 (n=41 and n=40), day 180 (n=9 and n=40), and day 360 (n=0 and n=41); (B) CV-CV vaccinees with (PI) and without (noPI) prior SARS-CoV-2 infection after the latest vaccine dose on day 28 (n=27 and n=14), day 90 (n=24 and n=17), and day 180 (n=5 and n=4); (C) CV-CV-mRNA vaccinees with (PI) and without (noPI) prior SARS-CoV-2 infection after the latest vaccine dose on day 28 (n=0 and n=40), day 90 (n=0 and n=40), day 180 (n=2 and n=38), and day 360 (n=9 and n=34); (D) CV-CV-mRNA vaccinees with VBI (n=27) and without VBI (noVBI) (n=56). The slopes were modelled with piecewise linear mixed models with each individual’s peak antibody level as a random effect, introducing knots at day 28, 90, and 180 after last vaccination. The black dots in panels (A, C and D) indicate the time of VBI occurrence. The estimated antibody values for each time point and the slopes between timepoints can be found in Table S6 Abbreviations: PI, prior infection; VBI, Omicron vaccine breakthrough infection

The third (mRNA-1273) vaccine dose was associated with a higher anti-S IgG day-28 (peak) titer compared to primary CV vaccination (LSM3.64 log_10_ BAU/mL [95%CI3.51, 3.78] vs 2.33 log_10_ BAU/mL [2.16, 2.49]). (Fig. 3, Table S5). Anti-S IgG titers declined significantly between day 28 and 90 after the third (mRNA-1273) vaccine dose (1.47% decline per day [1.23% to 1.71%]; *p* < 0.001) and reached a plateau between day 90 and 360 (p > 0.05). VBI occurrence was associated with a subsequent anti-S IgG titer increase of 0.13% per day (-0.01% to 0.27%; *p* = 0.076), although this association was of borderline significance, compared to stable anti-S IgG titers in participants without VBI (0.03% increase per day during day 181–360 [-0.19% to 0.26%]; *p* = 0.766).

## Discussion

This real-world study in Indonesian healthcare workers who received a heterologous CoronaVac-mRNA-1273 prime-boost vaccine regimen investigated the magnitude and longevity of humoral immune responses, induced by sequential viral antigen exposure. These exposures comprised prior SARS-CoV-2 infection before vaccination, a heterologous ancestral-strain mRNA vaccine booster, and/or subsequent Omicron BA.1/BA.2 breakthrough infection.

There is evidence indicating that previous SARS-CoV-2 infection enhances the potency, breadth and durability of the humoral response to primary vaccination [14]. However, our study findings among individuals vaccinated with two doses of CoronaVac, and other studies among recipients of mRNA vaccines, suggest that this effect may be short-lived, with decreases in spike-specific IgG and nAbs observed regardless of prior infection [15]. Moreover, evidence suggests that vaccination elicits more robust SARS-CoV-2 antibody responses, in terms of specificity, breadth and maturation against viral variants, as well as cellular immune responses, compared to natural infection [15, 16].

In contrast to several previous studies indicating rapid decline in antibody responses within six months after two-dose CoronaVac priming [8–10], our study observed sustained presence of B.1.1.529 and BA.2 nAbs up to 180 days after the second CV dose. This persistence may be attributed to potential unrecorded virus exposure during the BA.1/BA.2 wave in February–March 2022 among our study cohort.

The heterologous mRNA-1273 vaccine booster elicited robust peak titers of anti-S IgG and nAbs against variants of concern, consistent with earlier findings on heterologous mRNA booster strategies [17]. However, between day 28 and 90 post-booster, we observed a rapid decline in B.1.1.529 nAbs, resulting in loss of seropositivity in seven out of 40 (17%) participants, and anti-S IgG titer waning at a rate of 1.47% per day. These findings align with a study among HCWs in Israel who received a homologous mRNA (BNT162b2, BioNTech/Pfizer) prime-boost regimen, also demonstrating that the third vaccine dose conferred greater durability than the second dose, with anti-S IgG waning at 1.32% per day, and that Omicron exhibited greater resistance to neutralization compared to wild-type and Delta variants [18].

During the Omicron BA.1/BA.2 epidemic wave in Jakarta in February and March 2022, one in three HCWs who received an mRNA-1273 booster experienced a breakthrough infection. Those who had breakthrough infections exhibited significantly lower titers of anti-Spike IgG, B.1.1.529 or BA.2 nAbs measured before the breakthrough compared to those who did not have a breakthrough. These findings in our population, who were primed with inactivated vaccines, corroborate and expand upon similar observations in populations receiving primary mRNA vaccine regimens in high-income settings [9, 19]. Our data reinforce the understanding that boosting with an ancestral-strain vaccine (such as mRNA-1273) after inactivated vaccine priming, does not confer adequate immune protection against emerging Omicron variants [20]. Nevertheless, none of the 27 participants who experienced breakthrough infections developed severe COVID-19, suggesting that a heterologous ancestral-strain vaccine booster dose after inactivated vaccine priming may still offer protection against severe disease from Omicron BA.1/BA.2. This finding is consistent with findings from observational studies in Singapore [21] Hong Kong [22] and Indonesia [23], which demonstrated that three doses of either homologous vaccination with inactivated or mRNA vaccines [21, 22], or heterologous vaccination with viral vector or mRNA booster after inactivated vaccine priming [23], provided protection against severe or fatal outcomes, despite breakthrough infections with B.1.1.529, BA.2 or BA.5 variants.

There are limited data concerning the longevity and breadth of the immune response elicited by CoronaVac and subsequent Omicron breakthrough infection. A previous prospective study in China among individuals who received three doses of CoronaVac found that spike-specific antibodies and cellular responses were present in the majority of vaccinated individuals twelve months after the third dose; furthermore, the study highlighted that infection with Omicron BA.5 significantly enhanced the magnitude, cross-reactivity, and persistence of serum neutralization, Fc-mediated phagocytosis, nasal spike-specific IgA responses, memory B cells, activated cTfh cells, memory CD4 + T cells, and memory CD8 + T cells, for both the ancestral strain and Omicron subvariants, compared to unvaccinated individuals [24].

Bivalent booster vaccines incorporating ancestral and Omicron strains have been deployed as a strategy to enhanced antibody magnitude and cross-reactivity against emerging variants [25, 26]. Data on the immune responses to bi- or multivalent vaccine boosters following priming with inactivated vaccines are warranted.

Several study limitations should be noted. First, despite regular screening, some breakthrough infections may have gone unnoticedor unreported, potentially leading to misclassification of participants [27]. Second, while accumulating evidence suggests that the anti-spike IgG and nAbs responses correlate with protection against infection, a definited correlate of long-term vaccine protection has yet to be established. Protection is likely influenced by additional factors such as cellular immunity, which is increasingly recognized for its role in preventing infection and severe disease [28, 29] Third, cross-reactivity against recently emerged Omicron subvariants, which exhibit increasing degrees of immune evasion [30], was not evaluated in this study. Lastly, the demographic characteristics of the study volunteers may not fully represent the broader Indonesian population, with a lack of representation of elderly individuals.

In conclusion, this longitudinal study offers valuable real-world insights into the longevity of anti-spike IgG and neutralising antibody responses to CoronaVac primary vaccination and subsequent ancestral-strain mRNA booster and/or Omicron breakthrough infection in Indonesia. Our findings suggest that, after two-dose inactivated vaccine priming, an mRNA-1273 booster dose did not offer immunity against Omicron BA.1/BA.2 subvariants, particularly among individuals with lower anti-Spike IgG and B.1.1.529/BA.2 nAbs. Current evidence indicates that ancestral-strain vaccine boosters, still widely used in many LMIC, may offer partial protection against severe disease. There is an urgent need to expand access to and coverage of vaccine boosters among vulnerable populations in Indonesia and other LMIC, to enhance protection against emerging, immune-evasiding SARS-CoV-2 variants.

### Supplementary Information

Supplementary Material 1.

## Data Availability

Data is provided within the manuscript or supplementary information files.
