# Supplementary material for: Anti-SARS-CoV-2 antibody dynamics after primary vaccination with two-dose inactivated whole-virus vaccine, heterologous mRNA-1273 vaccine booster, and Omicron breakthrough infection in Indonesian health care workers
Source: BMC Infect Dis. 2024 Aug 1;24:768. doi: 10.1186/s12879-024-09644-y (PMC11292869; doi:10.1186/s12879-024-09644-y)
Supplement: Supplementary file 1 — Supplementary Material 1. [file 12879_2024_9644_MOESM1_ESM.docx]

**Figure S1.** Flowchart of study design


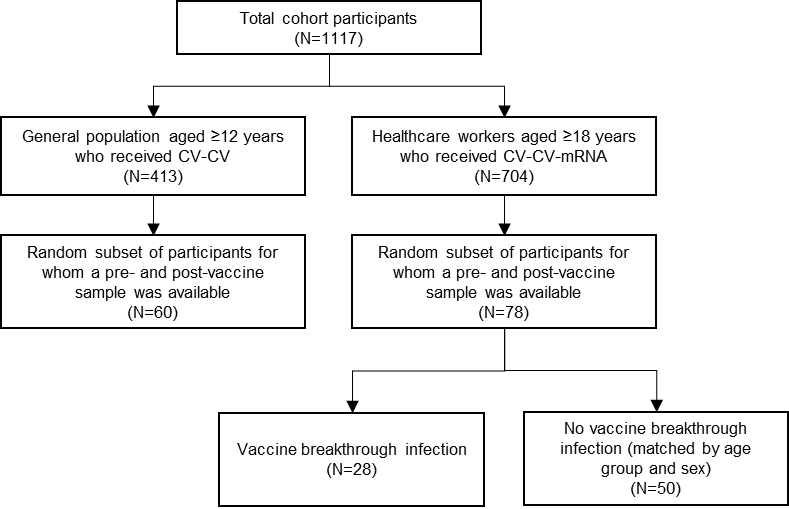


General population participants who received CoronaVac primary vaccination only (CV-CV; 2dose) and were followed up for up to 180 days post-vaccination.

Healthcare workers who received mRNA-1273 after CoronaVac primary vaccination (CV-CV-mRNA; 3dose) and were followed for up to 360 days post-vaccination.

A random subset of participants was selected for antibody analysis for whom data on pre-vaccine (day 0) and post-vaccine (day 28, 90, 180, 360, or vaccine breakthrough) were available.

Abbreviations: CV, CoronaVac; mRNA, mRNA-1273 (Moderna) vaccine.

**Table S1.** Number of participants included in the analysis per timepoint

| Sample available for analysis | **CV-CV vaccinees^1^**  **(N=60)** | | **CV-CV-mRNA vaccinees^2^**  **(N=78)** | |
| --- | --- | --- | --- | --- |
|  | **Prior infection (N=35)** | **No prior infection (N=25)** | **Prior infection (N=17)** | **No prior infection (N=61)** |
| Day 0 (pre-vaccine) | 35 | 25 | 16 | 26 |
| Day 28 | 27 | 14 | 0 | 40 |
| Day 90 | 24 | 17 | 0 | 40 |
| Day 180 | 5 | 4 | 2 | 38 |
| Day 360 | 0 | 0 | 9 | 34 |

^1^General population participants who received CoronaVac primary vaccination only (CV-CV; 2dose) and were followed up for up to 180 days post-vaccination.

^2^Healthcare workers who received mRNA-1273 after CoronaVac primary vaccination (CV-CV-mRNA; 3dose) and were followed for up to 360 days post-vaccination.

Abbreviations: CV, CoronaVac; mRNA, mRNA-1273 (Moderna) vaccine.

**Table S2.** Characteristics of all cohort participants and those included in the current analysis

| Characteristic | CV-CV vaccinees^1^ | | | CV-CV-mRNA vaccinees^2^ | | |
| --- | --- | --- | --- | --- | --- | --- |
|  | **Total**  **(N=413)** | **Included in analysis**  **(N=60)** | **P-value** | **Total**  **(N=704)** | **Included in analysis**  **(N=78)** | **P-value** |
| Sex |  |  |  |  |  |  |
| Female | 195 (47.2) | 22 (36.7) | 0.125 | 510 (72.4) | 56 (71.8) | 0.903 |
| Male | 218 (52.8) | 38 (63.3) |  | 194 (27.6) | 22 (28.2) |  |
| Age (median, IQR) – yrs | 32.0 (23.0-46.0) | 40.0 (26.5-53.0) | 0.089 | 31.0 (27.0-44.0) | 31.0 (26.0-48.0) | 0.899 |
| <30 | 188 (45.5) | 21 (35.0) |  | 301 (42.8) | 35 (44.9) |  |
| 30-49 | 137 (33.2) | 18 (31.7) |  | 308 (43.8) | 32 (41.0) |  |
| ≥50 | 88 (21.3) | 20 (33.3) |  | 95 (13.5) | 11 (14.1) |  |
| Any comorbidity | 193 (46.7) | 33 (55.0) | 0.231 | 346 (49.2) | 31 (39.7) | 0.115 |
| Obesity | 135 (32.7) | 17 (28.3) | 0.500 | 325 (46.2) | 27 (34.6) | 0.052 |
| Hypertension | 43 (10.4) | 7 (11.7) | 0.768 | 51 (7.2) | 7 (9.0) | 0.581 |
| Diabetes mellitus | 15 (3.6) | 6 (10.0) | 0.038 | 8 (1.1) | 2 (2.6) | 0.263 |
| Chronic lung disease | 7 (1.7) | 3 (5.0) | 0.122 | 2 (0.3) | 1 (1.3) | 0.271 |

P-value was derived from Chi^2^ or Fisher’s exact test.

^1^General population participants who received CoronaVac primary vaccination only (CV-CV; 2dose) and were followed up for up to 180 days post-vaccination.

^2^Healthcare workers who received mRNA-1273 after CoronaVac primary vaccination (CV-CV-mRNA; 3dose) and were followed for up to 360 days post-vaccination.

Abbreviations: CV, CoronaVac; mRNA, mRNA-1273 (Moderna) vaccine.

**Figure S2.** Timeline of median antibody titers measured during each calendar month, in the context of prior SARS-CoV-2 infection, administered vaccine doses and vaccine breakthrough infections


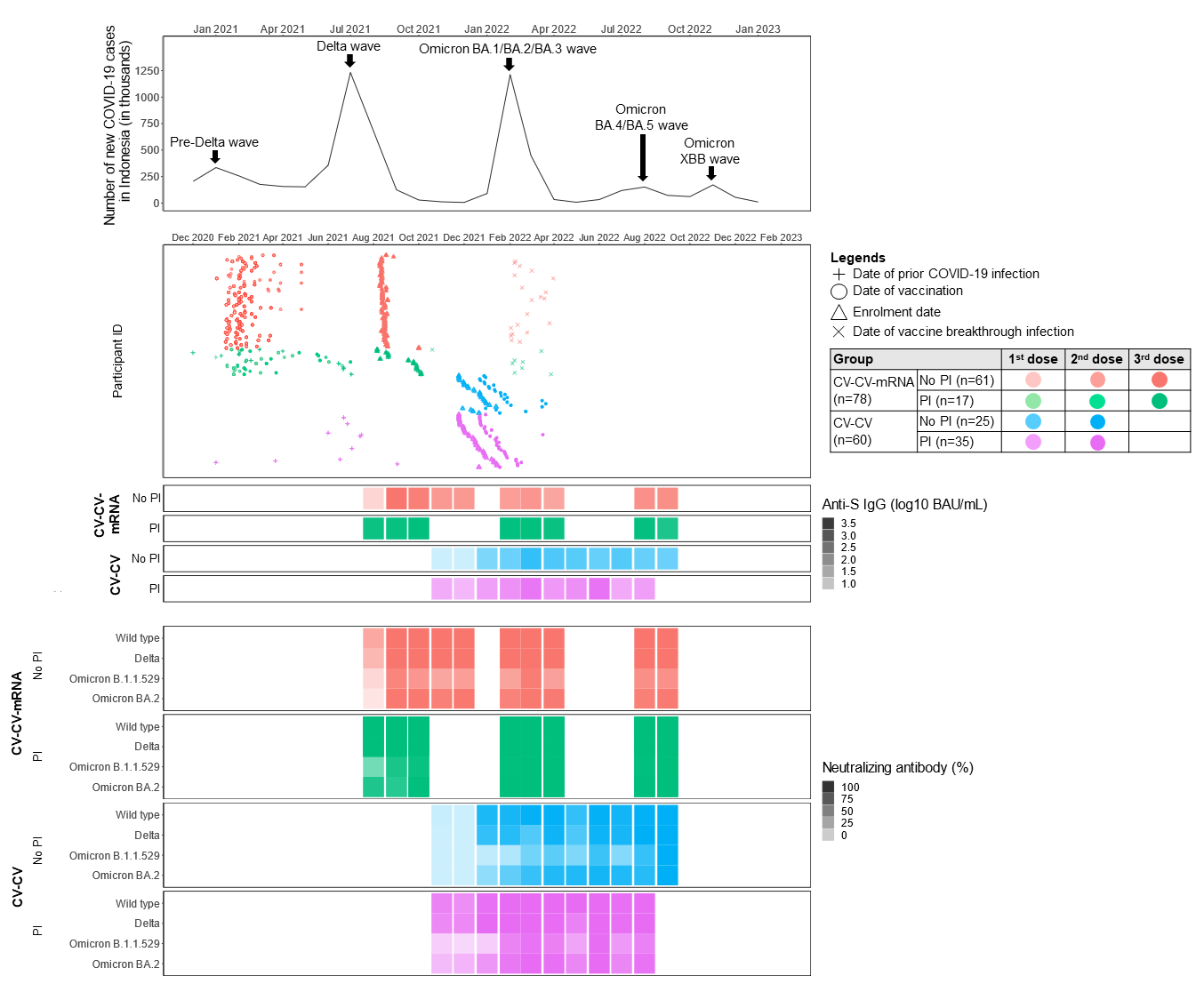


Top panel: reported numbers of new COVID-19 cases and the successive epidemic waves caused by SARS-CoV-2 variants in Indonesia from December 2020 through January 2023.

Centre panel: time plot of prior infection, study enrolment, vaccine doses administered, and Omicron breakthrough infection for each of the participants. Participants were enrolled from 6 August 2021 to 14 January 2022. CV-CV-mRNA vaccinees received their latest (third) vaccine dose from 6 August to October 2021 (red and green) and CV-CV vaccinees received their latest (second) vaccine dose from 22 December 2021 to 21 March 2022 (blue and purple). Prior SARS-CoV-2 infection (PI) before vaccination were mostly recorded during the pre-Delta or Delta epidemic waves. Vaccine breakthrough infections (red and green cross icons) were mostly recorded during the Omicron BA.1/BA.2 wave in February-March 2022, which was 4 to 6 months after the third (mRNA) vaccine dose.

Bottom panels: heat maps of median anti-spike IgG titers and neutralizing antibodies against wild-type, Delta, Omicron B.1.1.529, and Omicron BA.2 as measured during each calendar month. After receiving either a third (mRNA) dose (red and green dots) or a second (CoronaVac) dose (blue and purple dots), participants had a substantial and sustained increase of the IgG titers and neutralizing antibody titers against wild-type and Delta, whereas the increments of neutralizing antibodies against Omicron B.1.1.529 and BA.2 were much less apparent.

Abbreviations: CV, CoronaVac; mRNA, m-1273 (Moderna) vaccine; PI, prior SARS-CoV-2 infection.

**Figure S3.** Correlation of anti-S IgG titers and neutralizing antibodies against SARS-CoV-2 in CV-CV and CV-CV-mRNA vaccinees


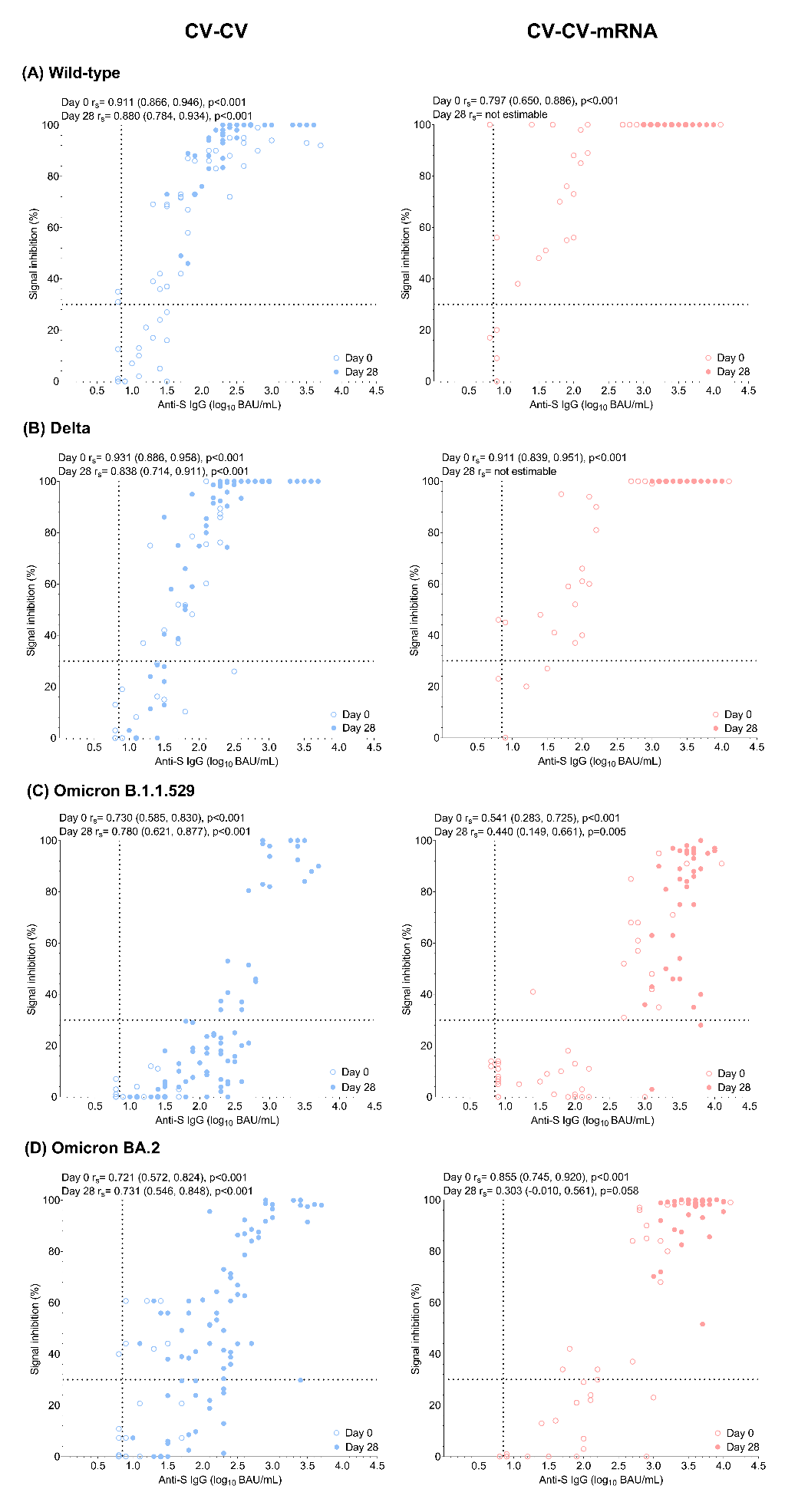


Figure shows the correlation between anti-S IgG titers and neutralizing antibodies against (A) Wild-type, (B) Delta, and (C) Omicron B.1.1.529 and (D) BA.2, stratified by vaccine doses and using Spearman's rank correlation test (r_s_) measured at pre-vaccine (day 0, blank dots) and post-vaccine visit (day 28, solid dots). The correlation coefficients between anti-S IgG titers and nAbs against (A) Wild-type and (B) Delta of CV-CV-mRNA vaccinees were not estimable as all participants had 100% signal inhibition on day 28.

Abbreviations: CV, CoronaVac; mRNA, mRNA-1273 (Moderna) vaccine; PI, prior SARS CoV-2 infection.

**Table S3.** Anti-S IgG titers and neutralizing antibodies in CV-CV-mRNA and CV-CV vaccinees

| **Timepoint** | **CV-CV vaccinees (N=60)** | | | **CV-CV-mRNA vaccinees (N=78)** | | | **P^2^** |
| --- | --- | --- | --- | --- | --- | --- | --- |
|  | **SP (n/N, %)** | **Median (IQR)** | **P**^1^ | **SP (n/N, %)** | **Median (IQR)** | **P**^1^ |  |
| **Anti-S IgG titers (log_10_ BAU/mL)** | | | | | | | |
| Day 0 | 50/60 (83.3) | 1.5 (0.9-2.2) | <0.001 | 40/42 (95.2) | 2.0 (0.9-2.9) | <0.001 | 0.014 |
| Day 28 | 41/41 (100) | 2.3 (2.1-2.9) | 0.120 | 40/40 (100) | 3.6 (3.4-3.7) | <0.001 | <0.001 |
| Day 90 | 41/41 (100) | 2.6 (2.3-3.2) | 0.099 | 40/40 (100) | 2.8 (2.4-3.0) | 0.062 | 0.346 |
| Day 180 | 9/9 (100) | 2.2 (2.0-2.4) | NA | 40/40 (100) | 2.6 (2.2-2.8) | 0.005 | 0.049 |
| Day 360 | NA | NA | NA | 43/43 (100) | 2.9 (2.6-3.1) | NA | NA |
| **Neutralizing antibodies (% signal inhibition)** | | | | | | | |
| **Wild-type** | | | | | | | |
| Day 0 | 35/60 (100) | 40.0 (2.7-86.0) | <0.001 | 32/42 (76.2) | 89.0 (38.0-100) | <0.001 | <0.001 |
| Day 28 | 41/41 (100) | 98.0 (88.0-100) | 0.238 | 40/40 (100) | 100 (100-100) | 0.338 | <0.001 |
| Day 90 | 40/41 (100) | 100 (96.0-100) | 0.214 | 40/40 (100) | 100 (100-100) | 0.093 | <0.001 |
| Day 180 | 9/9 (100) | 99.0 (78.0-99.0) | NA | 40/40 (100) | 100 (100-100) | 0.169 | <0.001 |
| Day 360 | NA | NA | NA | 43/43 (100) | 100 (100-100) | NA | NA |
| **Delta** | | | | | | | |
| Day 0 | 30/60 (100) | 33.0 (0.0-89.0) | <0.001 | 30/42 (71.4) | 61 (23-100) | <0.001 | 0.019 |
| Day 28 | 40/41 (100) | 100 (81.0-100) | 0.171 | 40/40 (100) | 100 (100-100) | 0.175 | <0.001 |
| Day 90 | 34/41 (100) | 97.0 (79.0-100) | 0.237 | 40/40 (100) | 100 (100-100) | 0.232 | <0.001 |
| Day 180 | 9/9 (100) | 100 (79.0-100) | NA | 40/40 (100) | 100 (100-100) | 0.476 | 0.061 |
| Day 360 | NA | NA | NA | 43/43 (100) | 100 (100-100) | NA | NA |
| **Omicron B.1.1.529** | | | | | | | |
| Day 0 | 7/60 (11.7) | 3.0 (0.0-18.0) | <0.001 | 15/42 (35.7) | 12.0 (5.0-52.0) | <0.001 | 0.002 |
| Day 28 | 18/41 (43.9) | 25.0 (9.0-90.0) | 0.088 | 38/40 (95.0) | 85.0 (52.0-96.0) | 0.014 | <0.001 |
| Day 90 | 32/41 (78.0) | 81.0 (47.0-89.0) | 0.254 | 33/40 (82.5) | 60.0 (38.0-88.0) | 0.355 | 0.160 |
| Day 180 | 9/9 (100) | 74.0 (35.0-91.0) | NA | 26/40 (65.0) | 59.0 (16.0-94.0) | 0.061 | 0.112 |
| Day 360 | NA | NA | NA | 38/43 (88.4) | 81.0 (46.0-92.0) | NA | NA |
| **Omicron BA.2** | | | | | | | |
| Day 0 | 34/60 (56.7) | 40.0 (3.0-61.0) | 0.002 | 17/42 (40.5) | 22.0 (0.0-84.0) | <0.001 | 0.184 |
| Day 28 | 29/41 (70.7) | 61.0 (28.0-94.0) | 0.002 | 40/40 (100) | 99.0 (95.0-100) | 0.001 | <0.001 |
| Day 90 | 36/41 (87.8) | 94.0 (82.0-100) | 0.400 | 40/40 (100) | 92.0 (81.0-98.0) | 0.342 | 0.103 |
| Day 180 | 9/9 (100) | 85.0 (65.0-96.0) | NA | 39/40 (97.5) | 89.0 (72.0-98.0) | 0.371 | 0.474 |
| Day 360 | NA | NA | NA | 43/43 (100) | 93.0 (71.0-98.0) | NA | NA |

Table shows IgG and neutralizing antibody (nAb) titers of participants who received CV-CV (2dose) or CV-CV-mRNA (3dose). Anti-spike IgG and nAbs against SARS CoV-2 wild-type, Delta, Omicron B.1.1.529 and BA.2 were measured at pre-vaccine, Day 0 (i.e. before the first dose in CV-CV vaccinees and before the third dose in CV-CV-mRNA vaccinees) and Day 28, 90, 180 (for all participants) and 360 days after vaccination for CV-CV-mRNA recipients. Values are expressed in median (IQR).

^1^Difference in antibody titers between the current and the subsequent timepoint (Dunn’s test, adjusted with Benjamini-Hochberg’s method for multiple comparisons).

^2^Difference in antibody titers between CV-CV and CV-CV-mRNA vaccinees for each timepoint (Dunn’s test).

Abbreviations: NA, not available; SP, seropositivity.

**Table S4.** Anti-S IgG titers and neutralizing antibodies in CV-CV and CV-CV-mRNA vaccinees with or without prior SARS CoV-2 infection

| **Timepoint** | **CV-CV vaccinees (N=60)** | | | | | | | **CV-CV-mRNA vaccinees (N=78)** | | | | | | |
| --- | --- | --- | --- | --- | --- | --- | --- | --- | --- | --- | --- | --- | --- | --- |
|  | **Prior infection (N=35)** | | | **No prior infection (N=25)** | | | **P^2^** | **Prior infection (N=17)** | | | **No prior infection (N=61)** | | | **P^2^** |
|  | **SP**  **(n/N, %)** | **Median (IQR)** | **P^1^** | **SP**  **(n/N, %)** | **Median (IQR)** | **P^1^** |  | **SP**  **(n/N, %)** | **Median (IQR)** | **P^1^** | **SP**  **(n/N, %)** | **Median (IQR)** | **P^1^** |  |
| **Anti-S IgG titers (Log_10_ BAU/mL)** | | | | | | | | | | | | | | |
| Day 0 | 35/35 (100) | 1.9 (1.5-2.6) | 0.001 | 15/25 (60.0) | 0.9 (0.8-1.2) | <0.001 | <0.001 | 16/16 (100) | 2.7 (2.0-3.0) | 0.411 | 24/26 (92.3) | 1.3 (0.9-2.2) | <0.001 | 0.002 |
| Day 28 | 27/27 (100) | 2.5 (2.3-3.3) | 0.147 | 14/14 (100) | 2.1 (1.9-2.3) | 0.281 | 0.001 | NA | NA | NA | 40/40 (100) | 3.6 (3.4-3.7) | <0.001 | NA |
| Day 90 | 24/24 (100) | 2.9 (2.6-3.4) | 0.105 | 17/17 (100) | 2.4 (2.1-2.8) | 0.394 | 0.004 | NA | NA | NA | 40/40 (100) | 2.8 (2.4-3.0) | 0.069 | NA |
| Day 180 | 5/5 (100) | 2.4 (1.9-3.0) | NA | 4/4 (100) | 2.2 (2.0-2.4) | NA | 0.356 | 2/2 (100) | 2.4 (2.4-2.4) | >0.999 | 38/38 (100) | 2.6 (2.2-2.8) | 0.003 | 0.267 |
| Day 360 | NA | NA | NA | NA | NA | NA | NA | 9/9 (100) | 2.8 (2.2-3.0) | NA | 34/34 (100) | 2.9 (2.7-3.1) | NA | 0.056 |
| **Neutralizing antibodies (% signal inhibition)** | | | | | | | | | | | | | | |
| **Wild-type** | | | | | | | | | | | | | | |
| Day 0 | 28/35 (80.0) | 83.0 (37.0-92.0) | <0.001 | 7/25 (28.0) | 1.0 (0.0-33.0) | <0.001 | <0.001 | 16/16 (100) | 100 (77.0-100) | 0.485 | 16/26 (61.5) | 55.5 (6.7-100) | <0.001 | 0.002 |
| Day 28 | 27/27 (100) | 100 (96.9-100) | 0.208 | 14/14 (100) | 88.5 (80.5-98.5) | 0.499 | 0.002 | NA | NA | NA | 40/40 (100) | 100 (100-100) | 0.333 | NA |
| Day 90 | 24/24 (100) | 100 (100-100) | 0.122 | 16/17 (94.1) | 99.0 (61.0-100) | 0.464 | 0.008 | NA | NA | NA | 40/40 (100) | 100 (100-100) | 0.069 | NA |
| Day 180 | 5/5 (100) | 99.0 (81.0-99.5) | NA | 4/4 (100) | 95.0 (71.5-99.7) | NA | 0.354 | 2/2 (100) | 100 (100-100) | 0.305 | 38/38 (100) | 100 (100-100) | 0.085 | 0.238 |
| Day 360 | NA | NA | NA | NA | NA | NA | NA | 9/9 (100) | 100 (100-100) | NA | 34/34 (100) | 100 (100-100) | NA | 0.077 |
| **Delta** | | | | | | | | | | | | | | |
| Day 0 | 26/35 (74.3) | 80.0 (28.5-99.6) | <0.001 | 4/25 (16.0) | 0.0 (0.0-17.6) | <0.001 | <0.001 | 15/16 (93.8) | 99.5 (66.0-100) | 0.414 | 15/26 (57.7) | 43.0 (0.0-95.5) | <0.001 | 0.002 |
| Day 28 | 27/27 (100) | 100 (98.7-100) | 0.055 | 13/14 (92.9) | 77.4 (50.9-92.0) | 0.435 | <0.001 | NA | NA | NA | 40/40 (100) | 100 (100-100) | 0.162 | NA |
| Day 90 | 23/24 (95.8) | 97.0 (93.2-100) | 0.340 | 16/17 (94.1) | 93.0 (15.5-100) | 0.294 | 0.070 | NA | NA | NA | 40/40 (100) | 100 (100-100) | 0.256 | NA |
| Day 180 | 5/5 (100) | 100 (76.5-100) | NA | 4/4 (100) | 94.0 (74.5-100) | NA | 0.394 | 2/2 (100) | 99.5 (99.0-100) | 0.467 | 38/38 (100) | 100 (100-100) | 0.426 | 0.249 |
| Day 360 | NA | NA | NA | NA | NA | NA | NA | 9/9 (100) | 100 (97.5-100) | NA | 34/34 (100) | 100 (100-100) | NA | 0.333 |
| **Omicron B.1.1.529** | | | | | | | | | | | | | | |
| Day 0 | 7/35 (20.0) | 13.0 (0.0-24.0) | <0.001 | 0/25 (0.0) | 0.0 (0.0-2.0) | 0.003 | <0.001 | 8/16 (50.0) | 23.0 (1.0-60.0) | 0.237 | 7/26 (23.1) | 11.5 (5.0-33.5) | <0.001 | 0.329 |
| Day 28 | 8/27 (29.6) | 37.4 (16.8-93.8) | 0.084 | 2/14 (14.3) | 8.1 (3.4-25.1) | 0.277 | 0.003 | NA | NA | NA | 38/40 (95.0) | 85.5 (51.0-96.0) | 0.013 | NA |
| Day 90 | 14/24 (58.3) | 84.0 (70.2-94.0) | 0.450 | 11/17 (64.7) | 56.0 (0.0-78.0) | 0.149 | <0.001 | NA | NA | NA | 33/40 (82.5) | 57.0 (38.0-88.5) | 0.341 | NA |
| Day 180 | 5/5 (100) | 81.0 (48.0-91.0) | NA | 4/4 (100) | 54.5 (35.0-93.5) | NA | 0.309 | 1/2 (50.0) | 54.0 (8.0-100) | 0.398 | 25/38 (65.8) | 56.0 (15.7-94.0) | 0.067 | 0.378 |
| Day 360 | NA | NA | NA | NA | NA | NA | NA | 8/9 (88.9) | 84.0 (34.5-90.0) | NA | 30/34 (88.2) | 78.0 (49.7-92.7) | NA | 0.488 |
| **Omicron BA.2** | | | | | | | | | | | | | | |
| Day 0 | 27/35 (77.1) | 53.3 (36.0-69.8) | 0.032 | 7/25 (28.0) | 7.3 (0.0-41.0) | 0.017 | <0.001 | 11/16 (68.8) | 38.0 (24.5-84.7) | 0.105 | 6/26 (23.1) | 0.5 (0.0-27.2) | <0.001 | 0.006 |
| Day 28 | 21/27 (77.8) | 73.0 (38.8-96.6) | 0.003 | 8/14 (57.1) | 32.4 (16.5-62.5) | 0.110 | 0.012 | NA | NA | NA | 40/40 (100) | 99.1 (94.5-99.9) | <0.001 | NA |
| Day 90 | 22/24 (91.7) | 100 (87.2-100) | 0.214 | 14/17 (82.4) | 85.0 (37.0-93.5) | 0.269 | 0.002 | NA | NA | NA | 40/40 (100) | 91.9 (79.8-98.3) | 0.320 | NA |
| Day 180 | 5/5 (100) | 91.0 (61.5-96.5) | NA | 4/4 (100) | 83.0 (60.7-96.2) | NA | 0.451 | 2/2 (100) | 77.0 (54.0-100) | 0.443 | 37/38 (97.4) | 88.9 (72.3-96.7) | 0.360 | 0.402 |
| Day 360 | NA | NA | NA | NA | NA | NA | NA | 9/9 (100) | 96.0 (61.5-98.5) | NA | 34/34 (100) | 92.0 (73.2-97.2) | NA | 0.377 |

Table shows anti-spike IgG and neutralizing antibody (nAb) titers of participants who received CV-CV (2dose) or CV-CV-mRNA (3dose) with or without prior SARS CoV-2 infection. Anti-spike IgG and nAbs against SARS CoV-2 wild-type, Delta, Omicron B.1.1.529 and BA.2 were measured at pre-vaccine, Day 0 (i.e. before the first dose in CV-CV vaccinees and before the third dose in CV-CV-mRNA vaccinees) and day 28, 90, 180 (for all participants) and 360 days after vaccination for CV-CV-mRNA vaccinees.

^1^Difference in antibody titers between the current and the subsequent timepoint (Dunn’s test, adjusted with Benjamini-Hochberg’s method for multiple comparisons).

^2^Difference in antibody titers between CV-CV and CV-CV-mRNA vaccinees for each timepoint (Dunn’s test).

Abbreviations: NA, not available; SP, seropositivity**.**


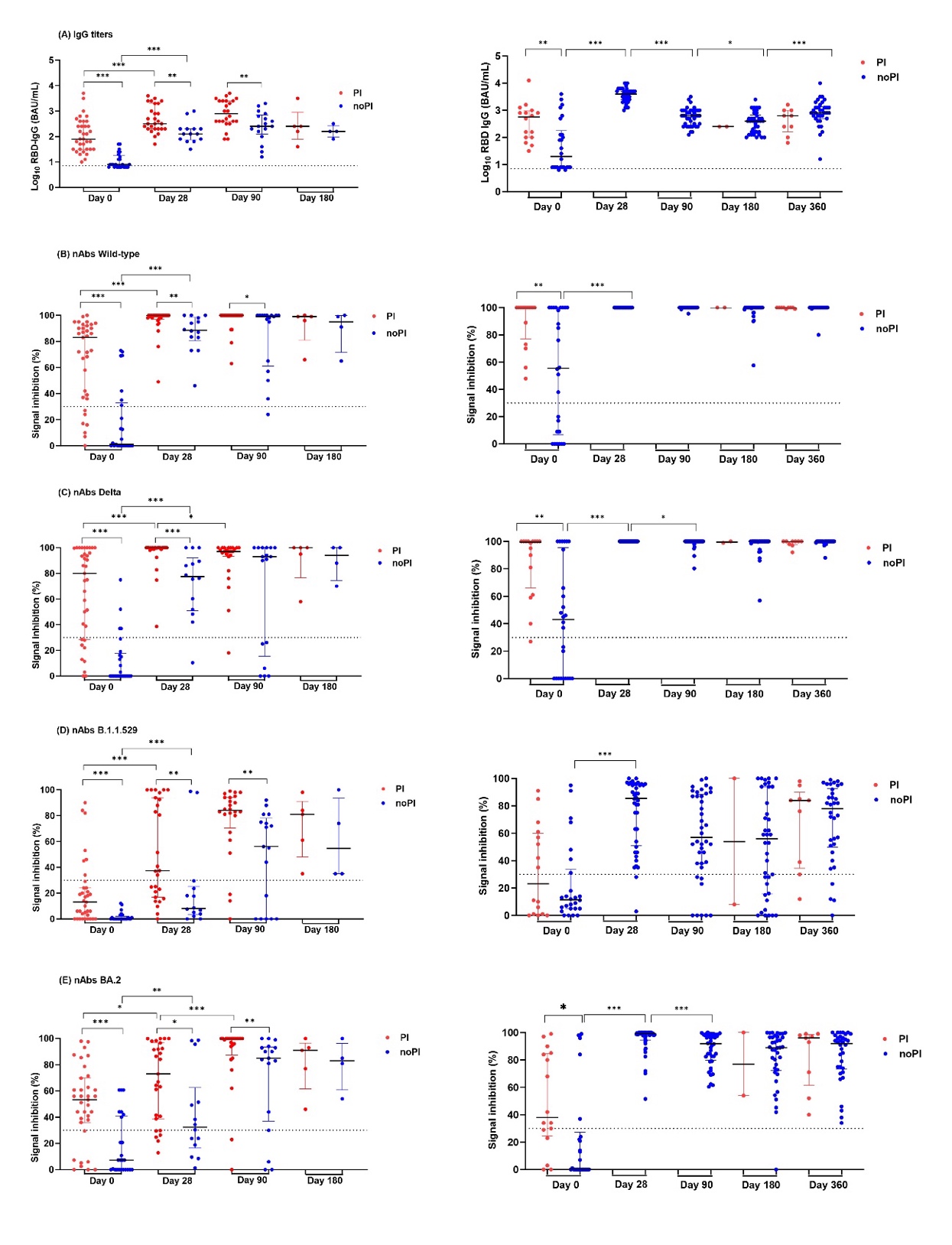
**Figure S4.** Antibody responses in CV-CV and CV-CV-mRNA vaccinees with or without a history of prior SARS-CoV-2 infection

**CV-CV**

**CV-CV-mRNA**

The figure shows data separately for CV-CV (left column) vaccinees and CV-CV-mRNA (right column) vaccinees with (PI, red dots) and without (noPI, blue dots) prior SARS-CoV-2 infection history.

PI was defined as, for CV-CV vaccinees, presence of anti-SARS-CoV-2 nucleocapsid protein, or a history of rapid antigen test or PCR-confirmed SARS-CoV-2 before first vaccine dose, and, for CV-CV-mRNA vaccinees, a history of rapid antigen test or PCR-confirmed SARS-CoV-2 infection before third dose vaccine.

Anti-S IgG titer (A) and neutralizing antibodies (nAbs) of SARS CoV-2 wild-type (B), Delta (C), Omicron B.1.1.529 (D) and BA.2 (E) were measured: i) Pre-vaccination (Day 0), i.e. before first dose in CV-CV vaccinees (PI n=35 and noPI n=25) and before third dose in CV-CV-mRNA vaccinees (PI n=16 and noPI n=26) and ii) Post-vaccination day 28 (CV-CV PI n=27 and noPI n=14; CV-CV-mRNA PI=0 and noPI n=40), day 90 (CV-CV PI n=24 and noPI n=17; CV-CV-mRNA PI n=0 and noPI n=40), day 180 (CV-CV PI n=5 and noPI n=4; CV-CV-mRNA PI n=2 and noPI n=38) and day 360 day (CV-CV-mRNA PI n=9 and noPI n=34).

Dashed line represents the IgG titer [≥7.1 BAU/mL = 0.85 log_10_ BAU/mL] and cut-off value for presence of neutralizing antibodies [signal inhibition >30%]. P-values were derived from Kruskal-Wallis H followed by Dunn’s post-hoc tests, adjusted by Benjamini-Hochberg method for multiple comparisons. *, p<0.05; **, p<0.01; ***, p<0.001.

Abbreviations: BAU, binding antibody units; CV, CoronaVac; mRNA, mRNA-1273 (Moderna); PI, prior infection.

**Table S5.** Anti-S IgG and neutralizing antibodies in CV-CV-mRNA vaccinees with or without Omicron vaccine breakthrough infection

| **Timepoint** | **VBI (N=27)** | | **NoVBI (N=56)** | | **P^4^** |
| --- | --- | --- | --- | --- | --- |
|  | **Median (IQR)** | **P** | **Median (IQR)** | **P** |  |
|  |  |  |  |  |  |
| Pre-VBI (Nov 21-Mar 22) | 2.6 (2.1-2.8) | 0.010^1^ | 2.7 (2.4-3.0) | NA | 0.029 |
| VBI (Feb-Mar 22) | 2.8 (2.6-3.0) | 0.206^2^ | NA | NA | NA |
| Post-VBI (Feb-Sep 22) | 2.9 (2.7-3.1) | 0.006^3^ | 2.8 (2.4-3.0) | 0.359^3^ | 0.112 |
| **Neutralizing antibodies (% signal inhibition)** | | | | | |
| **Wild-type** | | | | | |
| Pre-VBI (Nov 21-Mar 22) | 100 (97.4-100) | 0.058^1^ | 100 (100-100) | NA | 0.001 |
| VBI (Feb-Mar 22) | 100 (100-100) | 0.411^2^ | NA | NA | NA |
| Post-VBI (Feb-Sep 22) | 100 (100-100) | 0.036^3^ | 100 (100-100) | 0.254^3^ | 0.472 |
| **Delta** | | | | | |
| Pre-VBI (Nov 21-Mar 22) | 99.6 (93.3-100) | <0.001^1^ | 100 (100-100) | NA | <0.001 |
| VBI (Feb-Mar 22) | 100 (100-100) | 0.174^2^ | NA | NA | NA |
| Post-VBI (Feb-Sep 22) | 100 (99.7-100) | 0.028^3^ | 100 (100-100) | 0.026^3^ | 0.369 |
| **Omicron B.1.1.529** | | | | | |
| Pre-VBI (Nov 21-Mar 22) | 39.0 (3.0-55.5) | <0.001^1^ | 65.0 (43.8-91.2) | NA | 0.004 |
| VBI (Feb-Mar 22) | 94.0 (59.0-99.0) | 0.491^2^ | NA | NA | NA |
| Post-VBI (Feb-Sep 22) | 90.0 (79.5-97.0) | <0.001^3^ | 59.0 (38.0-92.5) | 0.360^3^ | 0.013 |
| **Omicron BA.2** | | | | | |
| Pre-VBI (Nov 21-Mar 22) | 84.4 (67.0-92.4) | <0.001^1^ | 92.7 (81.8-98.0) | NA | 0.011 |
| VBI (Feb-Mar 22) | 98.0 (95.0-100) | 0.281^2^ | NA | NA | NA |
| Post-VBI (Feb-Sep 22) | 97.5 (91.5-99.3) | 0.007^3^ | 90.2 (74.7-96.9) | 0.170^3^ | 0.021 |

Table shows median anti-S IgG and neutralizing antibodies in CV-CV-mRNA vaccinees who had an Omicron vaccine breakthrough infection (VBI) (during BA.1/BA.2 wave in February-March 2022) and those who never had a VBI (noVBI). The VBI occurred at a median of 181 days (170-202) since third mRNA vaccine dose.

“Pre-VBI” represent the timepoint of the latest antibody measurement before VBI occurrence (median 55 (IQR33-88) days).

“Post-VBI” represents the timepoint of the first antibody measurement after VBI occurrence (median 101 (39-175) days).

^1^Difference between pre-VBI and VBI antibody titers (Dunn’s test, adjusted with Benjamini-Hochberg’s method for multiple comparisons).

^2^Difference between VBI and post-VBI antibody titers (Dunn’s test, adjusted with Benjamini-Hochberg’s method for multiple comparisons).

^3^Difference between pre-VBI and post-VBI antibody titers (Dunn’s test, adjusted with Benjamini-Hochberg’s method for multiple comparisons).

^4^Difference in antibody titers between CV-CV-mRNA and CV-CV vaccinees for each timepoint (Dunn’s test).

Abbreviations: NA, not available; VBI, vaccine breakthrough infection.

**Table S6.** Changes in anti-spike IgG titers after second and third vaccine dose

| **Subgroup** | **IgG titer**  **day 28** | **Rate of change**  **day 28-90** | **IgG titer**  **day 90** | **Rate of change**  **day 91-180** | **IgG titer**  **day 180** | **Rate of change**  **day 181-360** | **IgG titer**  **day 360** |
| --- | --- | --- | --- | --- | --- | --- | --- |
| **CV-CV vaccinees** | | | | | | | |
| Overall | 2.33  (2.16, 2.49) | **0.74**  **(0.39, 1.09)**  **p<0.001** | 2.80  (2.63, 2.96) | **-0.44**  **(-0.76, -0.11)**  **p=0.008** | 2.39  (2.10, 2.67) | NA | NA |
| PI | 2.49  (2.32, 2.68) | **0.84**  **(0.46, 1.23)**  **p<0.001** | 3.03  (2.84, 3.22) | **-0.64**  **(-1.04, -0.24)**  **p=0.002** | 2.44  (2.09, 2.79) | NA | NA |
| noPI | 2.11  (1.85, 2.38) | 0.52  (-0.05, 1.09)  p=0.074 | 2.45  (2.20, 2.69) | -0.25  (-0.70, 0.20)  p=0.272 | 2.20  (1.80, 2.60) | NA | NA |
| **CV-CV-mRNA vaccinees** | | | | | | | |
| **Overall** | 3.64  (3.51, 3.78) | **-1.47**  **(-1.71, -1.23)**  **p<0.001** | 2.71  (2.59, 2.84) | 0.03  (-0.22, 0.28) p=0.793 | 2.74  (2.58, 2.89) | 0.05  (-0.07, 0.18) p=0.379 | 2.85  (2.70, 2.99) |
| PI | NA | NA | NA | NA | 2.81  (2.52, 3.09) | -0.06  (-0.34, 0.22)  p=0.683 | 2.70  (2.36, 3.04) |
| noPI | 3.58  (3.45, 3.72) | **-1.52**  **(-1.75, -1.30)**  **p<0.001** | 2.63  (2.49, 2.76) | 0.27  (-0.01, 0.54) p=0.059 | 2.85  (2.68, 3.02) | 0.01  (-0.12, 0.14)  p=0.893 | 2.88  (2.72, 3.04) |
| VBI | 3.52  (3.33, 3.71) | **-1.26**  **(-1.62, -0.91)**  **p<0.001** | 2.74  (2.57, 2.90) | -0.02  (-0.32, 0.28)  p=0.877 | 2.69  (2.53, 2.85) | 0.13  (-0.01, 0.27)  p=0.076 | 2.92  (2.73, 3.12) |
| noVBI | 3.72  (3.53, 3.92) | **-1.66**  **(-1.96, -1.37)**  **p<0.001** | 2.69  (2.49, 2.89) | 0.06  (-0.46, 0.59),  p=0.814 | 2.77  (2.43, 3.12) | 0.03  (-0.19, 0.26)  p=0.766 | 2.80  (2.60, 3.00) |

Table shows the estimated marginal means [95% confidence intervals] binding antibody titers, expressed as binding antibody units per mL, between timepoints on day 28, 90, and 180 after last vaccination, for all participants, and the addition of day 360 for CV-CV-mRNA vaccinees. Rate of change was measured with linear mixed models of antibody titer slope between given time point, and expressed as percentage points change per day. PI was defined as, for CV-CV vaccinees, presence of anti-SARS-CoV-2 nucleocapsid protein, or a history of rapid antigen test or PCR-confirmed SARS-CoV-2 before first vaccine dose, and, for CV-CV-mRNA vaccinees, a history of rapid antigen test or PCR-confirmed SARS-CoV-2 infection before third dose vaccine. Omicron vaccine breakthrough infection (VBI) was defined as a PCR-confirmed SARS-CoV-2 infection at least 14 days after the third (mRNA) vaccine dose. VBIs occurred at a median of 181 (IQR170-202) days after the third (mRNA) vaccine dose (during the BA.1/BA.2 wave in February-March 2022).

Abbreviations: IQR, interquartile range; NA, not available; PI, prior infection; VBI, Omicron vaccine breakthrough infection
